# Impaired Control of Body Cooling during Heterothermia Represents the Major Energetic Constraint in an Aging Non-Human Primate Exposed to Cold

**DOI:** 10.1371/journal.pone.0007587

**Published:** 2009-10-23

**Authors:** Jeremy Terrien, Alexandre Zahariev, Stephane Blanc, Fabienne Aujard

**Affiliations:** 1 Mécanismes Adaptatifs et Évolution, UMR CNRS/MNHN 7179, Brunoy, France; 2 Institut Pluridisciplinaire Hubert Curien, DEPE UMR CNRS 7178, Université de Strasbourg, Strasbourg, France; Universidad Europea de Madrid, Spain

## Abstract

Daily heterothermia is used by small mammals for energy and water savings, and seems to be preferentially exhibited during winter rather than during summer. This feature induces a trade-off between the energy saved during daily heterothermia and the energy cost of arousal, which can impact energy balance and survival under harsh environmental conditions. Especially, aging may significantly affect such trade off during cold-induced energy stress, but direct evidences are still lacking. We hypothesized that aging could alter the energetics of daily heterothermia, and that the effects could differ according to season. In the gray mouse lemur (*Microcebus murinus*), a non-human primate species which exhibits daily heterothermia, we investigated the effects of exposures to 25 and 12°C on body composition, energy balance, patterns of heterothermia and water turnover in adult (N = 8) and aged animals (N = 7) acclimated to winter-like or summer-like photoperiods.

Acclimation to summer prevented animals from deep heterothermia, even during aging. During winter, adult animals at 12°C and aged animals at 25°C exhibited low levels of energy expenditure with minor modulations of heterothermia. The major effects of cold were observed during winter, and were particularly pronounced in aged mouse lemurs which exhibited deep heterothermia phases. Body composition was not significantly affected by age and could not explain the age-related differences in heterothermia patterns. However, aging was associated with increased levels of energy expenditure during cold exposure, in concomitance with impaired energy balance. Interestingly, increased energy expenditure and depth of heterothermia phases were strongly correlated.

In conclusion, it appeared that the exhibition of shallow heterothermia allowed energy savings during winter in adult animals only. Aged animals exhibited deep heterothermia and increased levels of energy expenditure, impairing energy balance. Thus, an impaired control of the heterothermic process induced high energy costs in the aging mouse lemur exposed to cold.

## Introduction

The high volume/surface ratios and low capacities for energy storage [1] observed in small mammals have been associated with the expression of daily heterothermia as this strategy efficiently reduces the costs of normothermia [for review], [see 2,3]. Daily heterothermia expression, which mainly consists in a reduction in metabolic rate [4], is particularly useful when environmental conditions become harsh, e.g. when food is scarce or ambient temperatures (Tas) are low. This process can thus be seen as an evolved strategy that allows animals to increase their fitness in changing environments with limited resources.

Such adaptive response has been evidenced in the gray mouse lemur (*Microcebus murinus*), a small nocturnal primate originating from Madagascar, and mainly improves energy and water savings. Indeed, free-living mouse lemurs using daily heterothermia are able of reducing their energy expenditure and water turnover by almost 10 and 30%, respectively [5], [6]. However, it is likely that the trade-off between the energy saved during daily heterothermia and the energetic cost of arousal impacts energy balance. In this manner, we recently observed from core temperature (Tc) recording and body mass changes indirect evidences that aging may significantly affect such trade-off during cold-induced energy stress in the gray mouse lemur [7]. Indeed, the loss of body mass during cold exposure despite higher expression of daily heterothermia observed in aged, but not adult gray mouse lemurs, suggests that deep daily heterothermia episodes in aged heterotherms increase rather than decrease daily energy expenditure [8], [9]. In fact, arousal from torpid state is a rapid and efficient phase that depends on activation of non-shivering thermogenesis [10]. The energy cost of arousal can be significant and can account for up to 75% of the daily energy expenditure as shown in the California pocket mice [11]. Such high costs could lower the energy benefits of exhibiting deep heterothermia and imbalance the energy budget in the aging mouse lemur. Direct evidences are, however, still lacking.

Overall the gray mouse lemur is an ideal model to better understand how daily heterothermia impacts energy balance when environmental and physiological conditions vary. In thermoneutral conditions, the mouse lemur performs heterothermia and exhibit robust daily rhythms in Tc [12]. Many of its biological functions, i.e. body composition, reproduction, energy metabolism and thermoregulatory capacities, are synchronized on seasonal variations of photoperiod. As a long-day breeder, the mouse lemur becomes sexually active and looses body mass at the end of winter by anticipating the breeding season [13]. By contrast, the gonadal activity spontaneously regresses and animals reduce their metabolism to spare energy by anticipating the winter season. In addition to daily heterothermia, this nocturnal species has developed different behaviors (gregarism, nesting in buffered holes) to limit energy expenditure [14], [15]. These mechanisms are particularly enhanced during winter, a season during which water and food are scarce and Ta can reach very low values. In captive gray mouse lemurs, there are many evidences of strong interactions between the daily rhythm of Tc and photoperiod [16], ambient temperatures [7], [14], [16] and food availability [14], [17], suggesting that modulation of Tc is a key adaptive mechanism in this species. Finally, the mean life span of the mouse lemur in captivity is about 8 years [18], and aging is associated in this primate with a decrease in amplitude of the seasonal variations in body mass, gonadal hormones [19], melatonin [20], and DHEA-S [21], [22].

The study was performed to investigate the extent to which the deep daily heterothermia expression observed in aged mouse lemur during cold exposure affects the energy budget of the animals. The underlying hypothesis is that deep daily heterothermia expression in aged animals is an energy costly, but obligatory process due to poor body insulation, as compared to the well described energy saving advantages in adults [5], [6]. As daily heterothermia appeared affected during cold exposure in aged animals acclimated to winter [7] but not in those acclimated to summer [23], we further tested the hypothesis that the impact of cold-induced stress on the energy budget will differ according to season.

## Methods

### Animals and housing conditions

All the gray mouse lemurs studied were males, born in the laboratory breeding colony of Brunoy (MNHN, France, license approval N° A91.114.1) and were pathogen free. General conditions of captivity were maintained constant**:** Ta (24–26°C), relative humidity (55%). Food (including fresh fruits and a milky mixture) and water were available *ad libitum*. In captivity, seasonal variations of physiological functions are entrained by alternating 6-month periods of summer-like long photoperiod (14 h of light/day) and winter-like short photoperiod (10 h of light/day) under artificial light (fluorescent tubes during the day and dim red light during the night). In the present study, male mouse lemurs were studied during both short (LD10/14) and long (LD14/10) daylengths, at least two months after the onset of each season. Physiological status of the animals was supposed to be stabilized. General conditions of captivity were applied and animals were maintained in social groups before and after experimenting. In the present study, adults (N = 8; mean age±SEM: 1.6±0.3 years, range: 1.0–2.4) and aged mouse lemurs (N = 7; mean age±SEM: 7.4±0.2 years, range: 6.4–8.3) were used for each season. These two age categories were based on the survival data measured in the breeding colony of Brunoy. Analysis of survival from 254 male mouse lemurs allowed to determine the mean life span (mean±SEM: 6.0±0.2 years), the mean life span of the 10% of the most long lived animals (10.0±0.2 years) and the observed maximal survival duration (12.0 years). All experiments were carried out in accordance with the European Communities Council Directive (86/609/EEC). All efforts were made to minimize nociception.

### Core temperature recording

Animals were maintained in climate chambers (Sanyo incubator MIR-253), in which air was filtered and light was provided by cool fluorescent lamps. Mouse lemurs were acclimated to the experimental device for 10 days at Ta = 25°C. They were then studied for 10 days at the reference Ta of 25°C and then exposed to a cold environment (10 days at 12°C). Core temperature (Tc) was measured using a telemetric device: a 2.5 g transmitter (TA10TA-F20, Data Science Co. Ltd, Minnesota, USA) was implanted under general anesthesia (Valium, 2 mg/100 g i.m.; Ketamine Imalgem, 10 mg/100 g i.p.) in the visceral cavity of the animals. Calibrations for each transmitter were provided by the manufacturer. Experiments were performed after at least 2 weeks of recovery. Mouse lemurs were isolated in individual cages provided with branches and a wooden nest. A receiving plate (RPC-1, Data Science Co Ltd, Minnesota, USA) located in the cage permitted to register the data given by the transmitter. The Tc (in °C) was recorded every 10 minutes. Data were computed by a software (Dataquest Lab Pro v. 3.0, Data Science Co. Ltd, Minnesota, USA). Phase of Tc drop was delimited as followed: the onset of Tc decrease was determined as the first time point after which at least 3 successive bins of Tc decrease occurred; the end of Tc increase phase was determined as the time point occurring before the 3 successive bins of stable Tc. Then, we divided the analysis of Tc drop in two parts: first, parameters were determined to take into account the duration and amplitude of Tc decrease (considered as the energy economy strategy phase); second, the same logic was followed for Tc increase (considered as the energy loss phase). Areas under the curve of Tc were calculated with Tc amplitudes in Y axis and durations in X axis, assuming that areas were assimilated as triangles. Thus, T_decr_, D_decr_ and A_decr_ respectively corresponded to the amplitude, duration and area of Tc decrease. By transposition, T_incr_, D_incr_ and A_incr_ respectively corresponded to the amplitude, duration and area of Tc increase. Both A_decr_ and A_incr_ were corrected by taking into account the daylength for allowing comparisons between seasons. The minimal Tc value, determined as Tc_min_, was also analyzed. All telemetric parameters were averaged for each thermal exposure.

### Body mass, caloric intake

Body Mass (BM) was measured every 2 days throughout the experiment. However, according to the effects of season and age on BM, only variations in BM were considered for further analysis. Body Mass Gain (BMG) was calculated as a mean ratio (in g/day) during the whole exposure durations. Animals were routinely fed a*d libitum* on a diet including fresh banana (393 kJ/100 g) and a homemade milky mixture containing baby cereals, eggs and milk (435 kJ/100 g). Daily caloric intake (CI) was calculated by subtracting the remaining food to the food mass given. CI was expressed in kJ according to the Diem table [24] and normalized to the BM of the animal (kJ/day*100 g BM). The evaporation-related loss was taken into account in the calculation of CI at each Ta.

### Body composition, daily energy expenditure and water turnover

Daily energy expenditure (DEE), fat mass (FM), fat-free mass (FFM) and water turnover were estimated over a period of 3 days by the Doubly Labeled Water (DLW) method already described [25], [26]. This protocol was applied on the 6^th^ day after the beginning of each thermal exposure in the expectation of an adjustment of physiological parameters to the new experimented Ta. Animals were weighed before the experiment to determine the dose of DLW to be injected. The urine collected at the 6^th^ day determined basal enrichment in ^2^H and ^18^O. Then animals were injected in the intra peritoneal cavity with 2.3 g/kg of a pre-mixed solution of 0.55 g/kg H_2_^18^O (Rotem Industries Ltd., Israel) and 0.15 g/kg ^2^H_2_O (Cambridge Isotope Laboratories, Andover, MA, USA) diluted in 10 g of NaCl 9 ‰. Isotopic equilibration in total body water (TBW) was determined from a blood sample collected at 1 hour post-dose from a sampling in the saphenous vein. Immediately after sampling, the capillaries were flame-sealed. The mouse lemur was then released inside its cage and urine samples were collected in cryogenically stable tubes 24, 48 and 72 hours after blood sampling. Blood and urine samples were respectively stored at 5°C and −20°C until analyses by isotope ratio mass spectrometry.

Water from serum and urine samples were extracted by cryo-distillation, as previously described [27]. 0.1 µL of water was reduced to hydrogen and carbon monoxide by reduction on a glassy carbon reactor held at 1400°C in an elemental analyzer (Flash HT; ThermoFisher Germany). Hydrogen and carbon monoxide gases were separated by a GC column held at 104°C coupled to a continuous-flow Delta-V isotope ratio mass spectrometer. Isotopic abundances of deuterium and 18-oxygen in hydrogen and carbon monoxide gazes were measured in quintuplicate and repeated if SD exceeded 2 and 0.5‰, respectively. All enrichments were expressed against International Atomic Energy Agency standards. CO2 production was calculated according to the single pool equation of Speakman [28]. DEE was calculated by the Weir's equation [29] using a food quotient of 0.86 estimated from the animal's diet. FFM was calculated from TBW by assuming hydration coefficient of 73.2%. FM was calculated by the difference of FFM from the body mass. Water turnover was calculated as previously described [28] and expressed in g/day. FFM and FM were expressed in g. DEE was expressed in kJ/day.

### Statistical analysis

According to the statistical design, dependent variables were analyzed with Generalized Linear Models (GLM) or with Linear Mixed Effect models (LME), built with the “nlme” function [30]. All dependent variables were checked for normality and homoscedasticity with models' residuals. T_decr_ and T_incr_, which were non-normal variables, were log-transformed to reach normality. LME were built by taking into account the inter-individual variability. Indeed, the effect of individual identity was declared as a random effect. In addition, since the same individuals were used at 12°C and 25°C, we allowed inter-individual variation to depend on temperature by declaring the slope of the effect of Ta as a random factor. Statistical models including the additive effects of season (two levels, winter *versus* summer), Ta (two levels, 12°C and 25°C) and age (two levels, adult *versus* old), and their interaction were constructed. Significance of effects were assessed by F-tests [31] with software R Version 2.6.0 [32]. The statistics presented therefore provide the formula of the final model, i.e. the model containing significant effects only. All the statistics are however presented in supplementary tables. Finally, spearman correlations were tested using software “Systat for Windows”. Values are presented in the text as means±SEM and statistics in which p<0.05 were considered to be significant.

## Results

### Body composition

Variations in mean BM, FFM and FM between 25 and 12°C are presented in Table 1. Adult animals acclimated to winter exhibited higher BM and FM than those acclimated to summer. In adult animals, mean BM and FM values increased between 25 and 12°C in winter and remained quite stable in summer (Table 1). Seasonal variations in BM and FM were slight in aged mouse lemurs, and cold exposure did not induce major change in body composition. No significant variations in FFM could be evidenced, whatever the age and the season.

**Table 1:** Parameters (means values±SEM) representative of body composition measured at 25 and 12°C in adult and aged mouse lemurs acclimated to a winter-like or a summer-like photoperiod.

Age	Season	Ta	BM (g)	FM (g)	FFM (g)
**ADULT**	**Winter**	**25°C**	116.6±8.4	39.5±7.3	77.1±3.1
		**12°C**	125.2±13.3	46.4±10.4	78.8±7.1
	**Summer**	**25°C**	87.6±6.2	16.5±9.3	71.1±4.3
		**12°C**	88.5±7.4	14.8±7.2	73.7±2.0
**AGED**	**Winter**	**25°C**	106.7±5.6	30.9±4.7	75.8±6.7
		**12°C**	105.3±8.6	33.2±6.3	72.1±5.4
	**Summer**	**25°C**	97.4±9.7	20.0±6.7	77.4±5.6
		**12°C**	105.1±8.9	23.8±7.1	81.3±4.2
***LME:***	**Final model**		Age*Season*Ta	Age*Season*Ta	*NS*

LME were performed to assess for effects of season, age, Ta and their interactions. The final models, i.e. containing significant effects only, are provided for each parameter. NS: non significant.

*Parameters abbreviations: BM = Body mass; FM = Fat mass; FFM = Fat-free mass.*

### Water Turnover

**Figure 1:**
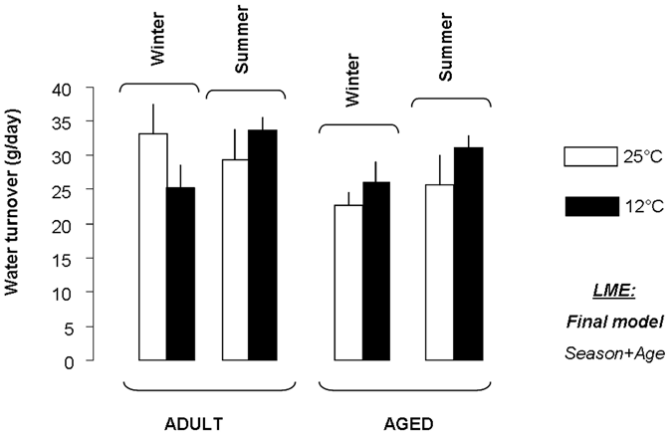
Strategy of water saving under cold exposure. Mean (±SEM) values (in g/day) of water turnover measured in adult and aged mouse lemurs acclimated to winter or summer and exposed to 25 and 12°C. Linear Mixed Effects (LME) models were performed to test the effects of Season, Ta, Age and their interactions. The final model, i.e. containing significant effects only, is provided.

Figure 1 represents variations in water turnover. In adult animals, no significant effect of cold exposure could be observed (Table S1), although water decreased by 19% between 25 and 12°C during winter. However, an effect of season was evidenced, particularly observable after cold exposure, with lower levels of water turnover during winter than during summer. Aged animals exhibited lower levels of water turnover than adults did, and levels were higher during summer than during winter. However, no effect of cold exposure could be evidenced.

### Daily heterothermia

**Figure 2:**
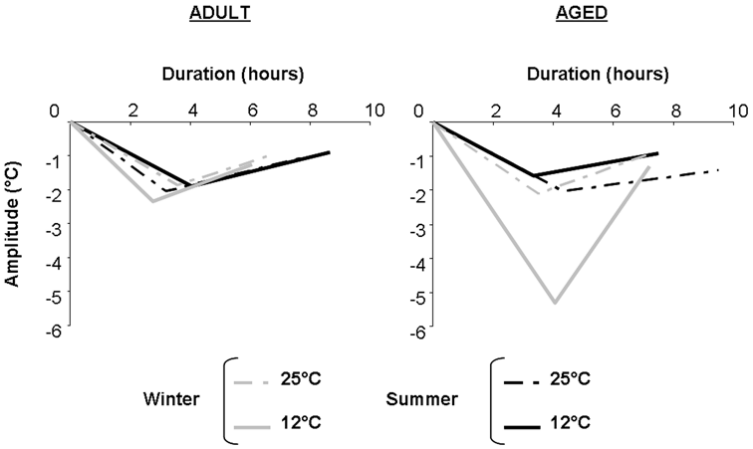
Variations in daily heterothermia patterns according to age, season and Ta. Schematic patterns of heterothermia phases observed in adult and aged mouse lemurs acclimated to winter (gray lines) or summer (black lines) and exposed to 25 (dotted lines) and 12°C (solid lines). The heterothermia phase patterns were determined from values of amplitudes and durations in Tc decrease and increase.

The variations of the parameters implied in the daily phase of Tc drop according to age, season and Ta together are exposed in Table 2 and Figure 2, while the statistics are exposed in Table S2.

**Table 2:** Parameters (means values±SEM) representative of both phases of Tc decrease and Tc increase observed at 25 and 12°C in adult and aged mouse lemurs acclimated to a winter-like or a summer-like photoperiod.

			Phase of Tc decrease			Phase of Tc increase		
Age	Season	Ta	Ddecr (hours)	Tdecr (°C)	Adecr (hours*°C)	Dincr (hours)	Tincr (°C)	Aincr (hours*°C)
**ADULT**	**Winter**	**25°C**	3.6±0.5	1.9±0.1	0.3±0.5	3.3±0.5	0.9±0.1	0.2±0.0
		**12°C**	2.8±0.2	2.3±0.3	0.3±0.1	3.3±0.5	1.1±0.1	0.2±0.0
	**Summer**	**25°C**	3.2±0.3	2.0±0.2	0.2±0.0	4.5±0.4	1.0±0.1	0.2±0.0
		**12°C**	4.0±0.4	1.9±0.2	0.3±0.0	4.6±0.5	1.0±0.2	0.2±0.0
**AGED**	**Winter**	**25°C**	3.5±0.5	2.1±0.4	0.4±0.1	3.7±0.4	1.2±0.2	0.2±0.0
		**12°C**	4.1±0.5	5.3±1.3	1.2±0.3	3.1±0.5	4.0±1.2	0.6±0.2
	**Summer**	**25°C**	4.3±0.5	2.0±0.1	0.3±0.1	5.2±0.6	0.6±0.0	0.1±0.0
		**12°C**	3.3±0.3	1.6±0.1	0.2±0.0	4.2±0.8	0.6±0.0	0.1±0.0
***LME:***	***Final model***		Age*Season*Ta	Age*Season*Ta	Age*Season*Ta	Age*Season*Ta	Age*Season*Ta	Age*Season*Ta

LME were performed to assess for effects of season, age, Ta and their interactions. The final models, i.e. containing significant effects only, are provided for each parameter.

*Parameters abbreviations: D_decr_ or D_incr_ = Duration of Tc decrease or Tc increase; T_decr_ or T_incr_ = Amplitude of Tc decrease or Tc increase; A_decr_ or A_incr_ = Areas under the Tc curve for Tc decrease or Tc increase.*

#### Tc decrease phase

The different parameters representative of the decrease phase in Tc were significantly affected by the interaction between effects of season, age and cold exposure. In adult animals, cold exposure induced a decrease in D_decr_ in adult animals during winter (from 3.6±0.5 to 2.8±0.2), whereas this parameter increased during summer (from 3.2±0.3 to 4.0±0.4). T_decr_ remained stable in adult animals after cold exposure during summer, whereas a slight increase from 1.9±0.1 to 2.3±0.3°C was observed in winter. Since BM interfered with Tc_min_ levels (LME: F_(1, 18)_ = 3.3, p = 0.08), BM values were integrated as covariate in the statistical analysis. Minimal Tc values differed according to the interaction between season and cold exposure (LME: F_(1, 19)_ = 7.03, p = 0.02). At 25°C, adult animals exhibited average Tc_min_ values of 35.6±0.2°C in winter and 35.8±0.1°C in summer. After cold exposure, theses values were slightly lowered and were on average 34.6±0.3 and 35.9±0.2°C in winter and summer, respectively. A_decr_, which integrated both modulations of D_decr_ and T_decr_, remained quite stable throughout the experiment in adult animals.

In aged animals, the variations in D_decr_ contrasted with those observed in adult mouse lemurs. In fact, contrary to adults, a cold-induced increase was observed during winter (from 3.5±0.5 to 4.1±0.5) whereas D_decr_ decreased from 4.3±0.5 to 3.3±0.3 during summer. Aged animals also exhibited a very high value of T_decr_ during winter at 12°C in comparison to 25°C. This cold-induced increase was not observed during summer. Tc_min_ values were equal to 35.2±0.4°C during winter and 35.8±0.2°C during summer at 25°C. Effects of cold exposure on Tc_min_ levels were higher during winter than during summer, leading to values of 31.0±1.4 and 35.4±0.1°C, in winter and summer at 12°C, respectively. By integrating variations of D_decr_ and T_decr_, it appeared that levels of A_decr_ were 3-fold higher after cold exposure during winter whereas no variation was observed during summer. Also, a seasonal difference in A_decr_ levels appeared at 12°C in aged animals with a 6-fold decrease between winter and summer.

#### Tc increase phase

In adult animals, cold exposure did not modulate the values of D_incr_ in both winter and summer seasons, despite a significant effect of season. In fact, Tc increase lasted longer during summer than during winter, whatever the Ta. The amplitude of Tc increase remained quite stable in adult animals between season and Tas, although a slight increase of T_incr_ was observed between 25 and 12°C during winter. When integrating the two parameters of duration and amplitude of Tc increase, it appeared that A_incr_ remained stable in adult animals throughout the experiment.

The phase of Tc increase after reaching Tc_min_ was strongly modified in aged animals, whatever the considered parameter. Indeed, in aged mouse lemurs, lower values of D_incr_ were observed after cold exposure during both winter and summer. The amplitude of Tc increase was also strongly modified in aged mouse lemurs. In fact, cold exposure induced a strong increase in T_incr_ during winter whereas no change was observed during summer. Consequently, A_decr_ strongly increased between 25 and 12°C during winter whereas no significant variation was evidenced during summer.

### Energy intake

Parameters of energy intake are presented in Table 3. In adult mouse lemurs, cold exposure induced a decrease in BMG in winter, without significant change in CI (Table S3). In summer, BMG decreased between 25 and 12°C, even though a 19% cold-induced increase in CI was observed. In aged animals, cold exposure induced an increase in CI, whatever the season (Table S3). CI increased by 17% during winter and by 48% during summer. BMG decreased after cold exposure during winter and became negative, whereas it remained stable during summer between 25 and 12°C.

**Table 3:** Parameters (raw means values±SEM) representative of energy intake and energy expenditure measured at 25 and 12°C in adult and aged mouse lemurs acclimated to a winter-like or a summer-like photoperiod.

Age	Season	Ta	CI (kJ/day*100 g BM)	BMG (g/day)	DEE (kJ/day)
**ADULT**	**Winter**	**25°C**	131±17	1.2±0.5	97.1±15.7
		**12°C**	133±11	0.0±0.3	129.0±9.9
	**Summer**	**25°C**	145±12	0.2±0.2	88.6±8.9
		**12°C**	173±11	−0.4±0.3	135.0±8.2
**AGED**	**Winter**	**25°C**	114±12	0.4±0.2	66.1±14
		**12°C**	133±23	−0.4±0.4	133.3±11.2
	**Summer**	**25°C**	100±26	−0.3±0.5	83.9±5.8
		**12°C**	148±21	−0.2±0.3	143.0±12.0
***LME:***	***Final model***		Age*Ta	Season*Age	*Ta*

LME were performed to assess for effects of season, age, Ta and their interactions. The final models, i.e. containing significant effects only, are provided for each parameters.

*Parameters abbreviations: CI = Calorie Intake, BMG = Body Mass Gain, DEE = Daily Energy Expenditure.*

### Energy expenditure

**Figure 3:**
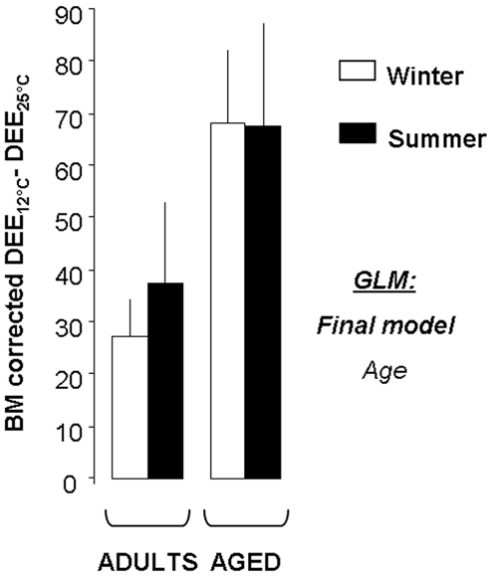
Increased levels of energy expenditure under cold exposure. Mean (±SEM) values (in kJ/day*100 g BM) representative of the cold-induced increase (12°C minus 25°C) in daily energy expenditure (DEE) levels corrected with body mass (BM) and measured between 25°C and 12°C in adult and aged mouse lemurs acclimated to winter or summer. Generalized Linear Models (GLM) were performed to test the effects of Season, Age and their interaction. The final model, i.e. containing significant effects only, is provided.

DEE values are presented in Table 3. In adult animals, DEE increased between 25 and 12°C, whatever the season (Table S3). When correcting the values of DEE with BM, it appeared that adult animals exhibited energy expenditure of 84±11 and 110±14 kJ/day*100 g BM at 25°C during winter and summer, respectively. The values increased to 108±10 and 156±16 kJ/day*100 g BM after cold exposure during winter and summer, respectively. Also, summer DEE values remained higher than those exhibited during winter, whatever the Ta. Figure 3 represents the differences between 12 and 25°C in DEE values corrected with BM. Cold-induced increase in DEE was similar between winter and summer in adult mouse lemurs (winter: 33% increase; summer: 52% increase).

In aged animals, DEE levels were 32% lower than in adult ones at 25°C during winter, although this was not evidenced statistically. When correcting DEE with BM values, aged animals exhibited levels of energy expenditure of 67±16 and 88±8 kJ/day*100 g BM at 25°C during winter and summer, respectively. The values increased to 136±10 and 149±23 kJ/day*100 g BM after cold exposure during winter and summer, respectively. As for adult animals, aged mouse lemurs exhibited higher values of DEE, even after cold exposure (Table S3). However, the cold-induced increase in DEE was higher in aged than in adult animals, as it is shown on Figure 3 (winter: 102% increase; summer: 70% increase).

**Figure 4:**
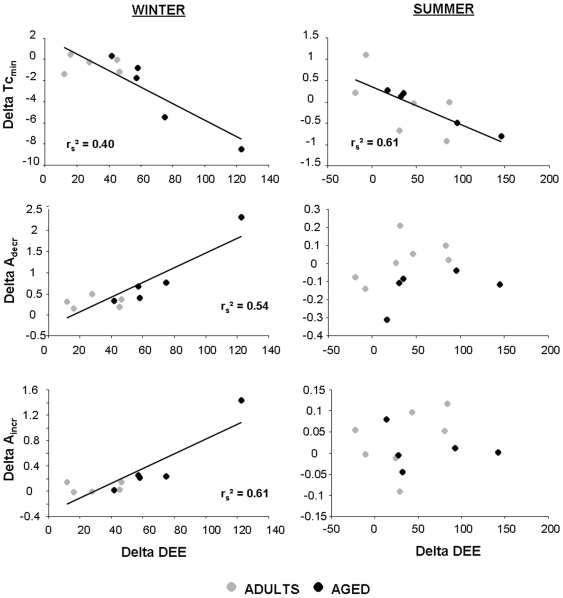
The deeper the heterothermia, the higher the energy expenditure. Spearman correlations observed between the cold-induced deltas (12°C minus 25°C) in daily energy expenditure (DEE) levels corrected with body mass and Tc_min_, A_decr_ and A_incr_ in adult and aged mouse lemurs acclimated to winter or summer.

Correlations between cold-induced differences in BM corrected values of DEE and parameters of Tc modulations were performed (Figure 4). Interestingly, mouse lemurs which exhibited the highest cold-induced decrease in Tc_min_ were those which exhibited the highest increase in BM corrected DEE, without distinction between winter and summer (winter: r_s_ = −0.63, p<0.1; summer: r_s_ = −0.78, p<0.05). However, correlations between DEE and A_decr_ or A_incr_ were only evidenced during winter but not during summer. Indeed, mouse lemurs acclimated to winter which exhibited the highest increase in A_decr_ or A_incr_ after cold exposure were those which exhibited the highest increase in DEE (A_decr_: r_s_ = 0.75, p<0.05; A_incr_: r_s_ = 0.78, p<0.05), even after removing the outlier (A_decr_: r_s_ = 0.70, p<0.05; A_incr_: r_s_ = 0.70, p<0.05). In addition, the increase in DEE was also positively correlated with the increase in T_incr_ (r_s_ = 0.83, p<0.05) and nearly with the increase in T_decr_ (r_s_ = 0.67, p<0.1) in mouse lemurs acclimated to winter. No such correlation could be evidenced between DEE and D_decr_ (r_s_ = 0.59, NS) or D_incr_ (r_s_ = −0.02, NS) during winter.

## Discussion

This study highlighted the effects of season and age on the energetics of daily heterothermia. First, adult animals succeeded in the maintenance of normothermia, whatever the season, suggesting different but efficient coping with low Tas. In contrast, cold exposure increased the depth of daily heterothermia with a high propensity in aged animals, this being related with increased levels of daily energy expenditure. Deep daily heterothermia expression in aged animals would thus consist in an energy costly, but obligatory process due to poor body insulation, as compared to the well known energy saving advantages in adults.

### Energetic implication in the use of heterothermia

Adult animals succeeded in maintaining their Tc in a narrow range after cold exposure during both winter and summer seasons. Regarding the DEE variations, the cold-induced increase in energy loss was only slight during both winter and summer. These results revealed that adult mouse lemurs were able of efficient coping with low Tas, whatever the season, suggesting different physiological responses according to season. In fact, shallow daily heterothermia is widely used in small mammal species for limiting the energy costs of normothermia [for review], [see 2,3]. Also, this privileged mechanism of energy and water savings is particularly enhanced during winter in the mouse lemur, such as it has already been evidenced in captive conditions [7]. In fact, during winter, season corresponding to food and water scarcity, mouse lemurs lower their metabolic rate and rest, leading thus to increased body mass. Also, body composition modulations are particularly well synchronized on seasonal variations of photoperiod in the mouse lemur [13], [33]. Such seasonal variations were confirmed in the present study. In fact, animals are able of fattening by preferentially allocating the energy intake to energy storage, a physiological mechanism explained by variations in some gut hormone levels [34]. This induced higher percentages of fat mass (FM) during winter than during summer, and this probably interfered with body heat production. Indeed, FM constitutes a huge reservoir of energy for fuelling thermogenesis processes such as non-shivering thermogenesis (NST) [35], [36]. This could explain the cold-induced decrease in body mass gain observed in the present study. In addition, daily heterothermia has been evidenced as an important mechanism for water savings [5], [6], [37]. In the present study, levels of water turnover did not show significant modulations after cold exposure. In fact, water was available *ad libitum* what maybe did not contribute to evidence the role of heterothermia in the reduction of water turnover during cold exposure. Nevertheless, water turnover decreased by 19% after cold exposure in adult animals acclimated to winter compared to summer. This suggested that the shallow heterothermia phases observed in adult mouse lemurs allowed water savings during cold exposure. Acclimation to summer prevented adult animals against deep heterothermia, even though animals were lean. This suggests that summer-acclimated animals relied on other mechanisms to keep their Tc in a normothermic range. Among those, the high levels of steroids, characteristic of the summer reproduction activity in this species [13], [38], are well-known as thermogenic agents [39]. More, Insulin-like Growth Factor type 1 (IGF-1) has been proposed to act in cold-induced thermogenesis processes in the rat [40], [41]. It appeared that IGF-1 levels were higher during summer [23] than during winter [7], what could also contribute to the seasonal difference in heterothermia depth.

### Aging alters the energy trade-off of daily heterothermia

Cold exposure induced strong modulations in Tc levels and the range of Tc was markedly increased, leading to deep heterothermia phases, particularly during winter. The decreased levels of Tc were correlated with increased levels of DEE, whatever the season. These results suggest that aged mouse lemurs did not exhibit efficient responses to limit the energy costs of cold response. Indeed, during summer, the hormonal thermogenesis advantage described in adult mouse lemurs could be lost during aging. In fact, steroid levels have been described to decrease with age in this species [19], [21], [22] and may account for increased DEE during normothermia maintenance. In contrast, during winter, the physiological response to cold would differ. In fact, at 25°C, aged animals acclimated to winter showed lower levels of DEE than adults, although this was not evidenced by the statistics. This could suggest that the shallow heterothermia observed at 25°C in aged animals was efficient for energy savings. More, aged mouse lemurs acclimated to winter exhibited lower levels of water turnover than adults at 25°C, also suggesting efficient water savings. This probably reflected that at 25°C, deeper heterothermia phases were observed in aged animals than in adult [7]. In fact, 25°C could already correspond to a value below the thermoneutral zone in aged animals and thus induce the necessity for water savings. However, these energy and water advantages were lost after cold exposure although heterothermia phases became very deep. In winter, the areas of Tc decrease and increase were markedly increased after cold exposure in aged mouse lemurs, this being mainly due to increased amplitudes of Tc decrease and increase. In fact, an interesting correlation linked the cold-induced increased levels of DEE with the increased phases of Tc drop. This is strongly supported by the fact that DEE was correlated with Tc amplitude but not with the duration of Tc increase. This suggested that the main energetic constraint could come from the amplification of daily heterothermia depth rather than from the slope of Tc decrease. The very low values of Tc observed in aged animals acclimated to winter would thus induce high rates of energy expenditure, which would contribute to the impaired energy balance during cold exposure. Such correlations suggested that aged animals, which exhibited the higher levels of DEE during cold exposure, were compelled to reach very low values of Tc even though they did not have the machinery necessary for efficient energy saving. We can hypothesize that the maintenance of body heat inside the organism could be impaired during aging. Since body composition did not appear impaired in the present study during aging, the decreased ability to keep body heat could be related to a decrease in cardiovascular performances, and more particularly to vasoconstriction capacities. Such age-related impairment is today well documented [42], [43], [44], [45] but has never been evidenced in the mouse lemur. Nevertheless, recent findings in the mouse lemur showed impaired capacities in dissipating fever during aging (unpublished data). The direct corollary for an age-related impairment in the ability of keeping body heat is higher costs for arousals. Indeed, torpor arousal essentially relies on brown adipose tissue non-shivering thermogenesis [10], [46], [47]. This thermogenesis has been shown to be active in the mouse lemur [48]. However, for maintaining a positive energy balance, this mechanism needs to expend less energy than the amount saved during Tc drop [49], [50], otherwise daily heterothermia becomes counterproductive [8], [9]. Regarding all these results, deep heterothermia phases did not appear as a beneficial compromise for energy and water economy in aged animals.

### Conclusions and perspectives

The present results raised novel issues on the modulations by season and age of the energetics of daily heterothermia. First, acclimation to summer appeared to prevent the mouse lemur, even during aging, from impaired energy balance when exposed to low ambient temperatures. During winter, the use of shallow heterothermia was usefull for energy savings in adult mouse lemurs only. Aged animals exhibited deeper heterothermia phases and impaired energy balance in response to cold. Moreover, the cold-induced increased energetic costs were strongly related to the amplitude of Tc decrease, which represents the major energetic constraint in aged individuals. The different results of this study pointed out a role for age-related deteriorations in cardiovascular capacities, which could compel aged animals to promote alternative strategies, such as behavioral ones, for maintaining a positive energy balance during cold exposure.

## Supporting Information

Table S1 Table of F statistics and p values after performing Linear Mixed Effects Models on parameters representative of body composition and water turnover. Statistical models including the additive effects of season (two levels, winter versus summer), Ta (two levels, 12°C and 25°C) and age (two levels, adult versus old), and their interactions were constructed. Statistics in which p≤0.05 were considered to be significant. *Parameters abbreviations: BM = Body Mass; FM = Fat Mass; FFM = Fat-Free Mass*.
(0.06 MB DOC)

Table S2 Table of F statistics and p values after performing Linear Mixed Effects Models on parameters representative of Tc decrease, Tc_min_ and Tc increase. Statistical models including the additive effects of season (two levels, winter versus summer), Ta (two levels, 12°C and 25 C) and age (two levels, adult versus old), and their interactions were constructed. Statistics in which p≤0.05 were considered to be significant. *Parameters abbreviations: D_decr_ or D_incr_ = Duration of Tc decrease or Tc increase; T_decr_ or T_incr_ = Amplitude of Tc decrease or Tc increase; A_decr_ or A_incr_ = Areas under the Tc curve for Tc decrease or Tc increase; Tc_min_ = Minimal Tc*.
(0.06 MB DOC)

Table S3 Table of F statistics and p values after performing Linear Mixed Effects Models on parameters representative of energy intake and energy expenditure. Statistical models including the additive effects of season (two levels, winter versus summer), Ta (two levels, 12°C and 25°C) and age (two levels, adult versus old), and their interactions were constructed. Statistics in which p≤0.05 were considered to be significant. *Parameters abbreviations: CI = Calorie intake; BMG = Body mass gain; DEE = Daily energy expenditure; BM = Body Mass*.
(0.06 MB DOC)
