# Supplementary material for: Impaired Control of Body Cooling during Heterothermia Represents the Major Energetic Constraint in an Aging Non-Human Primate Exposed to Cold
Source: PLoS One. 2009 Oct 23;4(10):e7587. doi: 10.1371/journal.pone.0007587 (PMC2761491; doi:10.1371/journal.pone.0007587)
Supplement: Table S1 — Table of F statistics and p values after performing Linear Mixed Effects Models on parameters representative of body composition and water turnover. Statistical models including the additive effects of season (two levels, winter versus summer), Ta (two levels, 12°C and 25°C) and age (two levels, adult versus old), and their interactions were constructed. Statistics in which p≤0.05 were considered to be significant. Parameters abbreviations: BM = Body Mass; FM = Fat Mass; FFM = Fat-Free Mass. (0.06 MB DOC) [file pone.0007587.s001.doc]

|  |  |  | **Body composition** |  | **Water Turnover** |
| --- | --- | --- | --- | --- | --- |
| **Effects** | **Parameters** | **BM** | **FM** | **FFM** |  |
| **Season** | **ddl** | 25 | 25 | 25 | 25 |
|  | **F** | 6.5 | 7.5 | 0 | 6 |
|  | **p** | 0.02 | 0.01 | 0.99 | 0.02 |
| **Ta** | **ddl** | 24 | 20 | 19 | 22 |
|  | **F** | 8.3 | 8.9 | 0.1 | 0.2 |
|  | **p** | 0.01 | 0.01 | 0.79 | 0.7 |
| **Age** | **ddl** | 25 | 25 | 25 | 25 |
|  | **F** | 2 | 0.1 | 0.1 | 4.1 |
|  | **p** | 0.17 | 0.71 | 0.75 | 0.05 |
| **Season*Ta** | **ddl** | 22 | 17 | 17 | 20 |
|  | **F** | 0.4 | 0.8 | 0.8 | 1.9 |
|  | **p** | 0.54 | 0.38 | 0.38 | 0.19 |
| **Season*Age** | **ddl** | 24 | 24 | 24 | 24 |
|  | **F** | 0.6 | 2.1 | 2.1 | 0.1 |
|  | **p** | 0.43 | 0.16 | 0.16 | 0.81 |
| **Age*Ta** | **ddl** | 22 | 18 | 17 | 20 |
|  | **F** | 4.5 | 3.5 | 0 | 1.1 |
|  | **p** | 0.04 | 0.08 | 0.88 | 0.3 |
| **Season *Age*Ta** | **ddl** | 21 | 17 | 16 | 19 |
|  | **F** | 4.9 | 4.3 | 1.2 | 1 |
|  | **p** | 0.04 | 0.05 | 0.3 | 0.34 |
